# High Time Resolution Analysis of Voltage-Dependent and Voltage-Independent Calcium Sparks in Frog Skeletal Muscle Fibers

**DOI:** 10.3389/fphys.2020.599822

**Published:** 2020-12-15

**Authors:** Henrietta Cserne Szappanos, János Vincze, Dóra Bodnár, Beatrix Dienes, Martin F. Schneider, László Csernoch, Péter Szentesi

**Affiliations:** ^1^Department of Biochemistry and Molecular Biology, School of Medicine, University of Maryland, Baltimore, Baltimore, MD, United States; ^2^Department of Physiology, Faculty of Medicine, University of Debrecen, Debrecen, Hungary

**Keywords:** skeletal muscle, frog, excitation-contraction coupling, ryanodine receptor, calcium-induced calcium release, membrane depolarization, calcium spark, caffeine

## Abstract

In amphibian skeletal muscle calcium (Ca^2+^) sparks occur both as voltage-dependent and voltage-independent ligand-activated release events. However, whether their properties and their origin show similarities are still in debate. Elevated K^+^, constant Cl^–^ content solutions were used to initiate small depolarizations of the resting membrane potential to activate dihydropyridine receptors (DHPR) and caffeine to open ryanodine receptors (RyR) on intact fibers. The properties of Ca^2+^ sparks observed under control conditions were compared to those measured on depolarized cells and those after caffeine treatment. Calcium sparks were recorded on intact frog skeletal muscle fibers using high time resolution confocal microscopy (x-y scan: 30 Hz). Sparks were elicited by 1 mmol/l caffeine or subthreshold depolarization to different membrane potentials. Both treatments increased the frequency of sparks and altered their morphology. Images were analyzed by custom-made computer programs. Both the amplitude (in ΔF/F_0_; 0.259 ± 0.001 vs. 0.164 ± 0.001; *n* = 24942 and 43326, respectively; mean ± SE, *p* < 0.001) and the full width at half maximum (FWHM, in μm; parallel with fiber axis: 2.34 ± 0.01 vs. 1.92 ± 0.01, *p* < 0.001; perpendicular to fiber axis: 2.08 ± 0.01 vs. 1.68 ± 0.01, *p* < 0.001) of sparks was significantly greater after caffeine treatment than on depolarized cells. 9.8% of the sparks detected on depolarized fibers and about one third of the caffeine activated sparks (29.7%) overlapped with another one on the previous frame on x-y scans. Centre of overlapping sparks travelled significantly longer distances between consecutive frames after caffeine treatment then after depolarization (in μm; 1.66 ± 0.01 vs. 0.95 ± 0.01, *p* < 0.001). Our results suggest that the two types of ryanodine receptors, the junctional RyRs controlled by DHPRs and the parajunctional RyRs are activated independently, using alternate ways, with the possibility of cooperation between neighboring release channels.

## Introduction

Calcium ion is a ubiquitous second messenger in many intracellular signaling pathways, but it also regulates highly specialized cellular processes such as contraction in muscle cells. Ca^2+^ sparks are the openings of a local cluster of ryanodine receptors (RyR) which are the Ca^2+^-release channels of the sarcoplasmic reticulum (SR; Wang et al., 2004). Sparks in amphibians are elementary events igniting global Ca^2+^ release during depolarization (Klein et al., 1997). Thus, the study of Ca^2+^ sparks provides useful information about the Ca^2+^ release process itself and the gating kinetics of RyR Ca^2+^ release channels.

Elementary calcium release events were first found in cardiac myocytes (Cheng et al., 1993), then in many other cell types as smooth (Nelson et al., 1995) and skeletal muscle (Tsugorka et al., 1995; Klein and Schneider, 2006). Morphology and frequency of calcium release events in various cell types, or from different species show marked alterations. One of the possible explanations could be the difference in the expression pattern of calcium release channels. In non-mammalian and embryonic mammalian skeletal muscle fibers two RyR isoforms coexist in almost equal amounts in contrast to fibers from adult mammals where the RyR1 isoform predominates (Sutko and Airey, 1996; Ogawa et al., 1999; Felder and Franzini-Armstrong, 2002). Interestingly mammalian skeletal muscle express the RyR3 isoform embryonically and certain muscles in adult, like diaphragm and soleus, and lower levels in abdominal muscles and tibialis anterior (Ogawa et al., 1999). The frog RyR isoforms (RyRα and RyRβ) are similar to mammalian RyR1 and RyR3, both in terms of their amino acid sequence and functional properties (Franzini-Armstrong and Protasi, 1997).

The α isoform is closely associated with the voltage sensor dihydropyridine receptors (DHPRs) located in the transverse (T-) tubule, and is voltage controlled. The β isoform is not connected with DHPRs, but even a small increase in intracellular calcium concentration can open it (Rodney and Schneider, 2003; Zhou et al., 2004; Pouvreau et al., 2007). The response to caffeine reportedly varies depending on animal species, muscle type, and age, with characteristically higher sensitivity and responsiveness reported on frog muscles than mammalian muscles which do not express RyR3 or just very low level (Ogawa et al., 1999; Rossi et al., 2001, Kashiyama et al., 2010).

Electron microscopic studies showed highly organized pattern of RYR receptors, called ‘feet’ on the SR membrane oriented toward the DHPR receptors in the T-tubule. Muscles expressing RyRβ/3 have additional clusters in the SR membrane. These are segregated from junctional RyRα/1s and are located in a parajunctional position (Felder and Franzini-Armstrong, 2002). This observation led to the hypothesis of two different mechanisms of RyR activation: a voltage-controlled intracellular calcium release through the RYRα/1s and the voltage-independent or calcium induced calcium release (CICR) pathway via the opening of RyRβ/3s (Rios and Pizarro, 1988; Franzini-Armstrong, 2004).

Previously, frog skeletal muscle Ca^2+^ sparks were shown to occur both in a voltage-dependent and voltage-independent, ligand-activated manner. Voltage-dependent sparks can be evoked experimentally by a subthreshold depolarization of a skeletal muscle fiber, and the frequency of Ca^2+^ sparks increases steeply with increasing depolarization (Klein et al., 1996). Voltage dependent activation of SR Ca^2+^ release in skeletal muscle is regulated by intramembrane voltage sensors, the dihydropyridine receptors (Franzini-Armstrong, 2004) in the transverse tubule membrane. The voltage dependence of intramembrane charge movement indicates that changes in membrane potential lead to the redistribution of these voltage sensors between intramembrane locations according to the Boltzmann relationship (Schneider and Ward, 2002). Voltage-independent sparks were ignited by endogenous ligands of RyRs via Ca^2+^-induced Ca^2+^ release mechanisms (Klein et al., 1996). Caffeine was used to activate RyRs and increase the frequency of the calcium release events on skeletal muscle fibers (Brum et al., 2000; Gonzalez et al., 2000a,b). In these studies increased frequency, higher amplitude and eccentricity was distinctive to the caffeine induced events.

Skeletal muscle fibers expressing both RyR isotype show a higher incidence of spontaneous calcium release events, than fibers missing the RyRβ/3 isotype. It was also found that spontaneous sparks remain visible in fully depolarized (voltage clamped to 0 mV) frog fiber, or blocking DHPRs (nifedipine, Klein et al., 1996, Cd^2+^/La^3+^ Perez et al., 2003). Although these applications could abolish the calcium movement through the voltage sensor DHPR, it was also demonstrated, that non-conducting DHPRs remain functional as voltage sensor for conformational EC coupling (Dayal et al., 2017).

Previous studies have also demonstrated that a chemical or mechanical skinning procedure results in a partial destabilization of the coupling between the DHPR–RyR that leads to the appearance of calcium release events (Kirsch et al., 2001; Zhou et al., 2003; Szappanos et al., 2005). Isaeva et al. (2005) found that the contents of the cytosol of skinned fibers were washed out and the number of the calcium release events changed consequently. Hollingworth et al. (2001) concluded in their studies that properties of spontaneous and voltage-activated sparks were similar in intact muscle fibers. They controlled membrane potential by using extracellular Ringer’s solutions with different potassium ion concentrations like as used in this study with some modification (see in “Materials and Methods”).

In our previous experiments using voltage clamped cut frog skeletal muscle fibers (Klein et al., 1996) we concluded that the amplitude of spontaneous sparks measured at the holding potential (–90 mV) was much smaller than that of voltage-dependent sparks during small depolarizations. Distribution of the amplitude of voltage-dependent sparks could be fitted by the sum of Gaussian functions with means being multiples of the unitary amplitude of the spontaneous sparks.

To re-examine the difference between voltage-dependent and independent sparks, as well as to characterize the voltage dependence of spark frequency, we have reinvestigated the properties of Ca^2+^ sparks performing a high time resolution analysis. To this end a Zeiss LSM 5 Live laser scanning confocal microscope system was used, with a maximum data acquisition speed up to 15.4 μs/line to record sparks in intact fibers isolated from frog toe muscle.

We found significant differences in the amplitude of the voltage-dependent and -independent sparks. Their spatial and temporal properties were different, similarly to the results of our previous experiments performed on cut fibers.

The novelty in our experiments are the high resolution data acquisition and the analysis. Using the high speed confocal microscopic technique with about 50 times faster acquisition rates at high resolution allowed us to study the spatial and temporal characteristics of sparks with minimal photo-toxicity. Part of this work was presented to the Biophysical Society (Vincze et al., 2013, 2014).

## Materials and Methods

### Fiber Preparation and Solutions

Frogs (*Rana pipiens*) were placed into crushed ice-water slurry for 30 min then killed by decapitation followed by spinal cord destruction according to protocols approved by the University of Maryland Institutional Animal Care and Use Committee. Toe muscles (*flexor digitorum brevis*) were removed, and individual muscle fibers were enzymatically isolated (8 mg/ml neutral dispase II, 1.3 mg/ml collagenase A, at 37°C, 1 h), in nominally calcium-free Ringer solution (in mM: 115 NaCl, 2.5 KCl, 1 MgCl_2_, 10 HEPES, pH 7.0). The separated, intact fibers were plated on extracellular matrix covered, glass bottom dishes, in Ringer solution containing 1.8 mM CaCl_2_. Before each experiments the cells were incubated with 20 μM Fluo-4 AM at room temperature for 30 min, followed by 20 min de-esterification.

To initiate voltage activated sparks in intact fibers we used high concentration potassium ion (K^+^) containing extracellular solutions (Table 1). Stepwise solution change with increased K^+^ content allowed us to reach small depolarizations between -70 and -60 mV while avoiding transient intracellular calcium level increase and subsequent contractions. The Goldman-Hodgkin-Katz equation was used to estimate the actual membrane potential in each K^+^ containing solutions. Methane sulfonic acid was used to keep the concentration of Cl^–^ constant in all solutions and so the ionic strength and the osmolality of the depolarization solutions.

**TABLE 1:** Composition of the external solution to induce different depolarizations.

**In mM**	**Ringer (−90 mV)**	**−70 mV**	**−65 mV**	**−60 mV**
NaCl	115	115	115	115
KCl	2.5	0	0	0
CaCl_2_	1.8	1.8	1.8	1.8
MgCl_2_	1	1	1	1
Hepes	10	10	10	10
K methane sulfonate	−	10.2	13.8	18.1

Caffeine in 1 mM concentration was used to increase the appearance of sparks.

Individual fibers were monitored and fibers with increased overall fluorescence or physical deformities following laser exposure or solution change were not used for spark recording. The basic spontaneous activity was also recorded for 10 and 5 fibers before introducing small depolarizations, or 1 mM caffeine, respectively. All measures were taken to avoid photo damage or photo bleaching therefore subsequent images were taken on the same fiber after repositioning. To avoid movement artifacts no local perfusion was used during recordings. This is demonstrated by combining all images in a time-series to show that structural elements did not move (Supplementary Figures 1C,D and Supplementary Video 1).

### High Speed Recording and Analysis of Ca^2+^ Sparks in Intact Fiber

The fibers were imaged using a Zeiss LSM 5 LIVE laser scanning confocal microscope (Zeiss, Oberkochen, Germany). Instead of imaging the specimen pixel by pixel, it scans along a whole line at the same time, obtaining high temporal resolution image at high acquisition speed. Two dimensional (x-y) image series were taken with speed of 30 or 60 frame per second (FPS), the pixel (line) acquisition time was 47.4 and 15.4 μs. The 512 × 512 pixel image corresponds to 106 μm × 106 μm image size in our experimental setting (pinhole 17 μm, objective: 63X water, NA: 1.2). Fluo-4 was excited with an Argon ion laser (at 488 nm), emitted light was collected through a beam-path filter and digitized at 12 bit. The images were normalized to events excluded baseline fluorescence (F_0_).

### Identification of Cell Structure and Calcium Sparks on x-y Images

**FIGURE 1:**
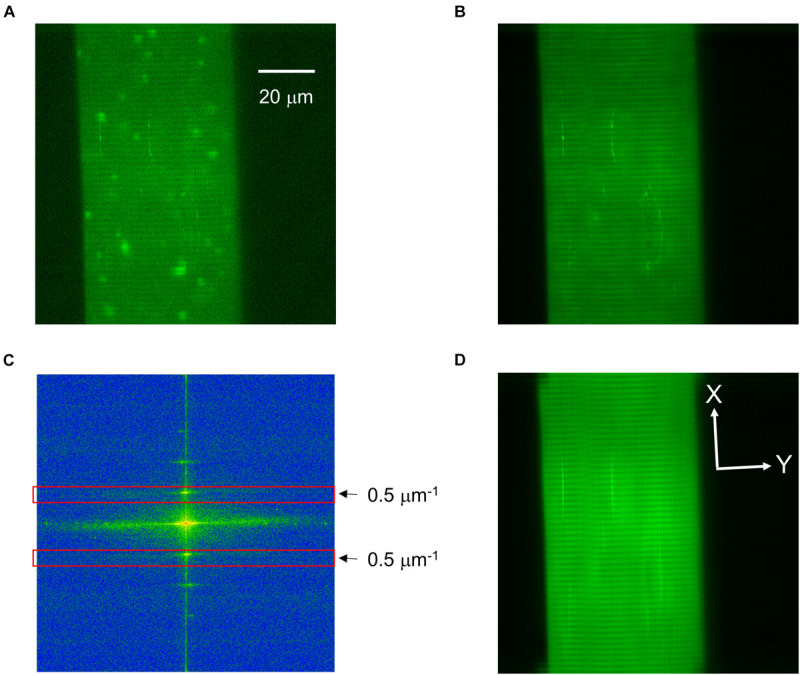
Identification of the cell structure in a representative x-y image. **(A)** An original x-y image of an intact frog skeletal muscle fiber loaded with Fluo-4 displaying calcium release events. **(B)** The picture of the basic fluorescence values of each pixel showing good signal-to-noise ratio for static image elements, such as Z-lines. **(C)** Fast Fourier transform showing the frequency spectrum of the image in panel **(B)**. At 0.5 μm^–1^ (and at its inverse, red rectangles) the sharp band corresponding to one sarcomere is clearly visible. **(D)** The result of the inverse Fourier transformation, where the Z-lines are excellently traceable. White lines perpendicular (X) and parallel (Y) to the Z-lines determine the directions of FWHMs. Scale of panels **(B,D)** is identical to that in panel **(A)**.

The x-y image sequences containing spontaneous elementary calcium release events (Figure 1A) were evaluated using a homemade computer program developed in Borland Delphi (Borland Software Co., Austin, TX, United States). The program allows full automatic analysis of the sequence of images without human intervention.

As the first step in image analysis, the baseline fluorescence value for each pixel of the image was determined. To obtain this value, the average fluorescence value of the points in the time domain for a given pixel that are below *Mean* + *NormCri⋅*σ values for all pixel was calculated, where Mean and σ correspond to the average and standard deviation of the fluorescence values of a given pixel in the time domain, respectively. The optimal value of *NormCri* was 1.1. As a next step, the components of the time series belonging to each pixel was determined by the single-dimensional stationary wavelet transformation (Szabo et al., 2010) and then a high pass filtering was applied. Finally, the changes in calcium levels with very slow kinetics not caused by spark formation was removed by dividing all the data of the time-series with the value of the 4th wavelet level (see Szabo et al., 2010, Figure 1).

The area belonging to the cell was determined from an image with good signal-to-noise ratio created from the baseline fluorescence values for each pixel (Figure 1B). The fluorescence of the cell-associated pixels was much higher than the fluorescence of the extracellular image points; the distribution of intensity was distinctly bimodal. The optimal cut between the two sets of pixels was determined with iterative method, then the area above the threshold was defined as the cell.

After the removal of all variable elements (i.e., calcium sparks) from the image series Z-lines became even more pronounced with some other constant features being present. The structural elements of the cell were determined by the Fourier transform of longitudinally oriented cell areas (Figure 1C). The values observed at 0.5 μm^–1^ corresponded to the expected (approximately 2 μm) sarcomere length. Except this peak and the center area of the frequency components all other coefficients were set to zero and an inverse Fourier transformation was performed. In the resulting picture only the components corresponding to the structural elements remained (Figure 1D). The Z-lines were determined by searching for the local maximum at columns. With this method the incidentally breaking Z-lines could have also been followed and the parameters of the events could be determined in directions perpendicular (X) and parallel (Y) to the Z-lines (Figure 1D).

The efficiency of Z-line identification was tested with images containing artificially created fiber structures. The inclination angle of fibers was determined from the orientation of Z-lines and was compared to the pre-defined creation angle. The developed method gave back almost identical inclination angles (Supplementary Figure 2).

**FIGURE 2:**
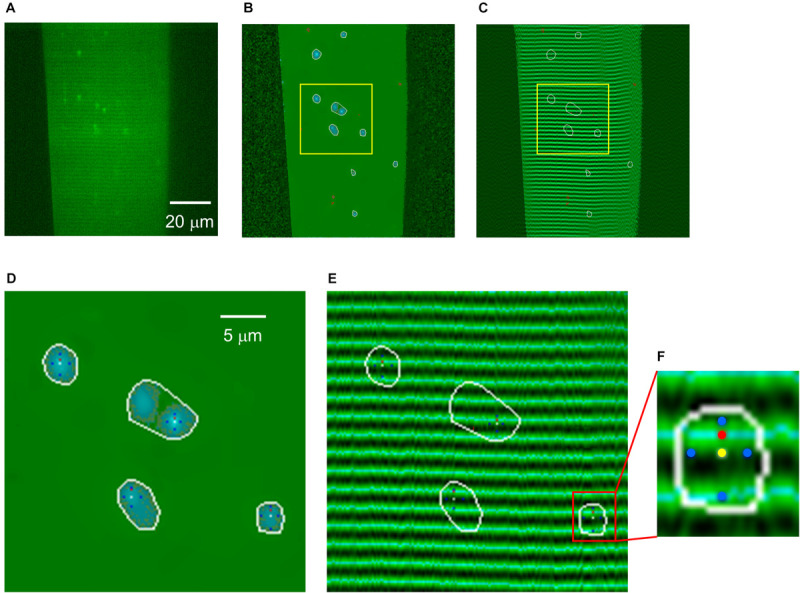
Generation of structure-free normalized image for spark identification and localization. **(A)** Original x-y image. **(B)** F/F_0_ image to show that the structure was removed and only the sparks are visible. **(C)** Fiber structure superimposed with areas of sparks. **(D)** Part of the image in **(B)** showing four events at high magnification. White curves encircle the areas corresponding to the identified events. Yellow dots present the position of the maxima, while the four blue dots in each event show the orientation of the two axis (perpendicular and parallel to the Z-lines) within the spark. Red pixels represent the position of the fluorescence maximum of Fluo-4, and thus the distance between yellow and red pixels was used to calculate the distance of the center of the spark from the nearest Z-line. **(E)** Same area as in panel **(B)** enlarged from panel **(C)** showing the superimposed Z-lines. The dark bands mark the triads at the Z-lines. **(F)** A spark enlarged from panel **(E)** showing the location of color dots described above. Light blue lines show the fluorescence maximum (i.e., the middle of a sarcomere).

Before identifying the ROIs and defining their parameters, high-efficiency Wavelet noise filtering (δ = 3; Szabo et al., 2010) was performed on each normalized image. To identify ROIs corresponding to elemental calcium release events a modified double-threshold method (Cheng et al., 1999) was used in the analysis (Figure 2). Contiguous areas with values over *Mean* + *LoCri⋅*σ that contained at least one pixel with a value greater than *Mean* + *HiCri⋅*σ were identified as events. The optimal value of *LoCri* and *HiCri* in our case was 1.7 and 2.8, respectively.

To test the method a spark free x-y image with sarcomeric structure and some higher intensity areas throughout the experiment, was merged with artificial Gaussian sparks with different amplitudes (Supplementary Figure 3A). Sparks were added to the image by convolving the function producing two-dimensional Gaussian curves with the image containing no sparks at random locations (minimal distance of sparks was 4 μm). Number of sparks per frame (30) and spatial parameters corresponded to the averages found under control conditions (Full-width at half maximum in the X direction, FWHM-X = 1.74 μm; Full-width at half maximum in the Y direction, FWHM-Y = 1.52 μm). The amplitude ranged between 0.15 and 0.6. The sensitivity of the wavelet based detection method was proven, all events with amplitude higher than 0.2 were identified (Supplementary Figure 3B). Events with amplitude of 0.2 were captured with around 0.6 sensitivity while events with smaller amplitude were lost. The positive predicted curve (Supplementary Figure 3C) was, however, very sharp, all events with amplitude higher than 0.15 were identified correctly. According to these findings events with amplitude less than 0.15 were excluded from all further evaluation. Due to the large number of sparks in x-y images analyzed, small sparks not found due to the chosen thresholds did not cause any significant drop in the analysis and considered as out of focus events.

Within all identified ROI a 3 × 3 area with the highest average amplitude was determined and the center was defined as the center of the event (Figure 2D). The difference between the fluorescence intensity of this point and the baseline was used to calculate the amplitude of the spark. The program also considered this point as focal point in the direction parallel and perpendicular to the Z-lines. Where the average intensity of three points reached half of the amplitude value of the spark, the program identified these points as the boundaries of FWHM-X and FWHM-Y. The distance for each event from the nearest Z-line was assessed by calculating the difference of the estimated half sarcomere length and the distance from the nearest fluorescence maximum (Figure 2E). The position of the center of the spark was identified on each consecutive image and was used to calculate the movement of the spark, if present, in the X and Y directions.

The effects of the amplitude on the other parameters of the spark were also tested. Images (*n* = 40) containing artificial sparks with different amplitudes (*n* = 30) were evaluated and the amplitude and FWHM in both directions were calculated. The program slightly under estimated the original amplitudes (Supplementary Figure 4A). Calculated FWHMs at both directions were the most accurate at 0.25 amplitude. At smaller amplitudes both FWHMs were under estimated, while at higher amplitudes they were overestimated (Supplementary Figures 4B,C). We also tested the effects of the orientation of fiber in the image on FWHMs. By limiting the ROI to the central part of the events, the FWHM values (Supplementary Figure 5) and eccentricity of sparks (Supplementary Figure 6B; see below) were not affected by fiber orientation.

Ca^2+^ spark frequency was calculated from the number of sparks during the recording period divided by the area of the fiber and the duration of the acquisition (number of sparks per area per time) at each conditions.

Signal mass (SM) was calculated as follows:

SM=1.206×(FWHM-X)×(FWHM-Y)×

(1) $$\left(\frac{\left(F\,W\,H\,M-X\right)}{2}+\frac{\left(F\,W\,H\,M-Y\right)}{2}\right),$$

where Amp is the amplitude, while FWHM-X and FWHM-Y have their usual meaning (Hollingworth et al., 2001). The spark was assumed to be a spherical 3D object with different X, Y and Z diameters. The diameter in the Z direction was estimated as the average of the X and Y diameter.

To assess the shape of the recorded spark a modified eccentricity value was calculated defined as:

(2) $$E=1-{\left(\frac{\mathrm{FWHM}-Y}{\mathrm{FWHM}-X}\right)}^{2},$$

Note that this is different from the classical definition of eccentricity for an ellipsis (see Supplementary Figure 6) to account for the case when FWHM-Y is greater than FWHM-X.

### Chemicals and Statistical Analysis

Chemicals, unless otherwise stated, were purchased from Sigma (St. Louis, CA, United States) and were of analytical grade.

Pooled data were expressed as mean ± standard error (SE) of the mean. The differences between control and treatments was assessed using one-way analysis of variance (ANOVA) and all pair wise multiple comparison procedures (Student-Newman-Keuls Method). F test was used to test the significance and a *p* value of less than 0.05 was considered statistically significant.

## Results

### Event Frequency and Parameters of Sparks in Different Conditions

**FIGURE 3:**
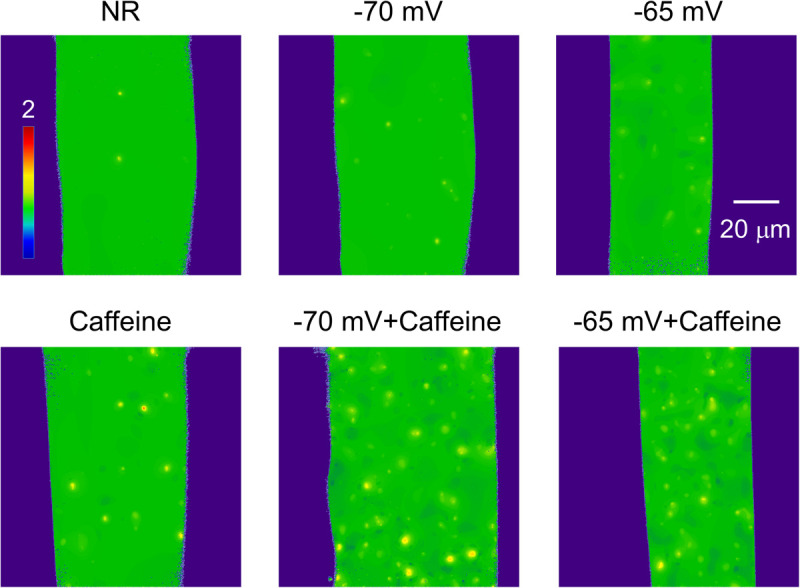
Normalized (F/F_0_) full size x-y images of intact frog skeletal muscle fibers bathed in Normal Ringer (NR), in the presence of 1 mM Caffeine (Caffeine), in depolarizing solutions corresponding to a calculated resting membrane potential of -70 and -65 mV, and supplemented with 1 mM Caffeine. All images were recorded with 30 FPS. Note how the number of sparks is changing with the different conditions.

**FIGURE 4:**
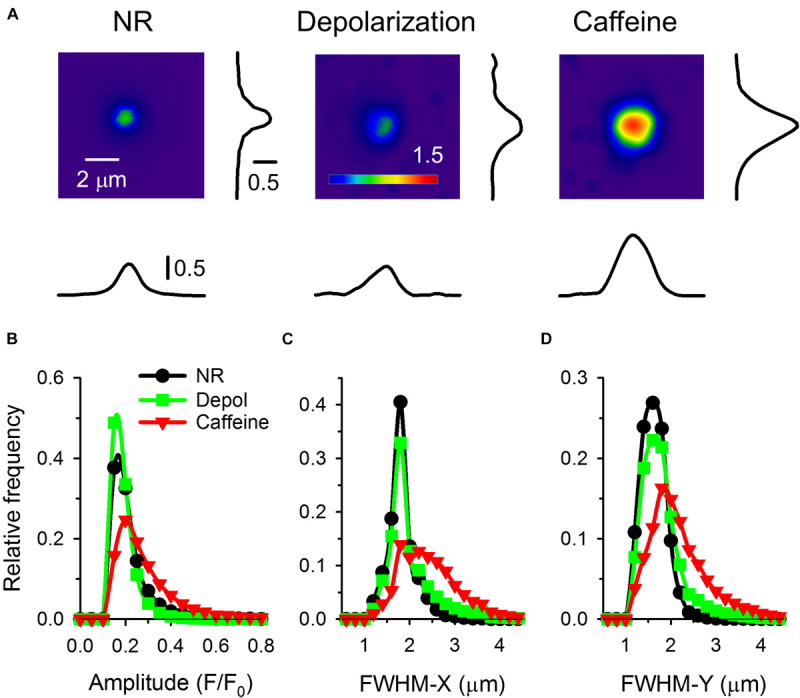
**(A)** Individual sparks on a larger scale. All images were recorded with 30 FPS. Next to the spark the spatial profile of F/F_0_ in X & Y direction (parallel and perpendicular to fiber axis) are presented. Panels **(B–D)** present the distribution histograms for the amplitude **(B)**, FWHM-X **(C)**, and FWHM-Y **(D)** under control conditions (black circles), on fibers depolarized to a calculated value of -65 mV (green squares), and in the presence of 1 mM caffeine (red triangles). Note how both depolarization and caffeine has shifted the distribution the higher values, especially visible in case of FWHM-X and FWHM-Y in the presence of caffeine from 2.5 μm to 4 μm.

Both caffeine treatment and depolarization increased the frequency, the amplitude, the spatial dimensions, and the signal mass of sparks in frog skeletal muscle fibers (Table 2 and Figures 3, 4). Using incremental depolarization steps, the highest frequency was found at -60 mV (Table 2). In the presence of 1 mM caffeine sparks occurred at higher frequency. Depolarization and caffeine treatment together led to even more sparks (Table 2). Simultaneously applied depolarization and caffeine gave the highest frequency at -65 mV. However, at this voltage both the amplitude, the spatial spread, and the signal mass were significantly reduced. This was likely due to the fact that the depolarization induced a partial inactivation of the voltage sensors (e.g., Huang, 1996) and the large frequency of sparks induced a partial depletion of calcium in the SR thus 1./greater depolarizations in the presence of caffeine were not explored in detail and 2./except for Table 2 the data for caffeine treatment at -65 mV were not combined with the other data and are presented in Supplementary Figures 7–9.

**TABLE 2:** Parameters of sparks in different conditions.

	**0 mM Caffeine**				**1 mM Caffeine**		
	**NR**	**−70 mV**	**−65 mV**	**−60 mV**	**NR**	**−70 mV**	**−65 mV**
Number of fibers	106	64	47	33	21	8	7
Number of events^§^	6164	55312	43326	21410	24942	16029	12391
Frequency (1/ms/mm^2^)	1.47 ± 0.30	24.10 ± 3.80*^#^	37.15 ± 4.35*	38.15 ± 8.25*	91.54 ± 21.36*^#^	115.28 ± 40.34*^#^	147.77 ± 60.40*^#^
Amplitude (F/F_0_)	0.188 ± 0.001	0.171 ± 0.001*^#^	0.164 ± 0.001*	0.173 ± 0.001*^#^	0.259 ± 0.001*^#^	0.305 ± 0.001*^#^	0.176 ± 0.001*^#^
FWHM-X (μm)	1.725 ± 0.005	1.836 ± 0.002*^#^	1.917 ± 0.003*	2.089 ± 0.004*^#^	2.342 ± 0.005*^#^	2.482 ± 0.005*^#^	2.150 ± 0.006*^#^
FWHM-Y (μm)	1.508 ± 0.004	1.587 ± 0.001*^#^	1.676 ± 0.002*	1.776 ± 0.004*^#^	2.081 ± 0.005*^#^	2.270 ± 0.006*^#^	1.845 ± 0.005*^#^
SM (μm^3^)	1.024 ± 0.009	1.138 ± 0.004*^#^	1.335 ± 0.007*	1.776 ± 0.004*^#^	4.354 ± 0.034*^#^	6.464 ± 0.062*^#^	2.085 ± 0.021*^#^

^§^All events with amplitude less than 0.1 and FWHM less than 1 and greater than 10 μm were discarded. *denotes significant difference from NR at *p* < 0.001. ^#^denotes significant difference from −65 mV without caffeine treatment at *p* < 0.001.

Calcium sparks detected on frog fibers were not symmetric in the spatial domain, their FWHM was smaller in the Y then in the X direction. Average FWHM in both directions was increasing with increased depolarization and further increased when caffeine was added (Table 2). The distribution histograms of spark parameters clearly demonstrate that both depolarization and caffeine increased the frequency of the larger sparks (Figures 4B–D), the effect was more pronounced for the latter. Since both depolarization and caffeine treatment elicit sparks with larger spatial widths, the calculated signal mass was also higher under these conditions (Table 2).

**FIGURE 5:**
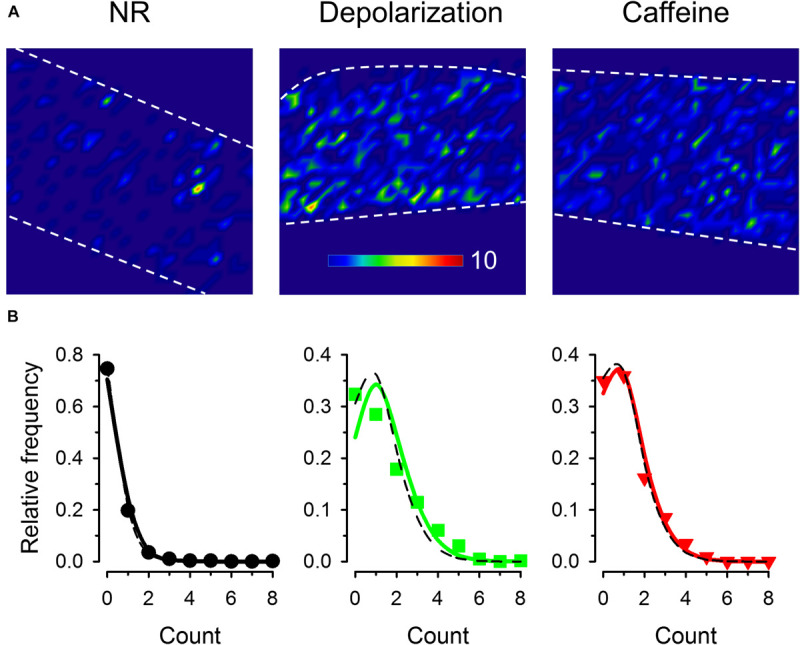
Identifying positions of spark occurrence in images. **(A)** Mesh plot of frequencies of spark occurrence (i.e., the number of sparks in the given region) in a 16 × 16 pixel voxels (generated from 60 consecutive images). All images were recorded with 30 FPS. **(B)** Frequency distribution of regions with different number of sparks. Solid line superimposed is the fitted Poisson distribution, while dashed black curves show the Poisson distribution generated with λ being equal to the average event numbers. The values for λ used to generate the dashed curves were 0.349, 1.42, and 1.12 while those obtained from the fits were 0.281, 1.19, and 1.04 for control conditions, for depolarized fibers, and in the presence of caffeine, respectively.

Representative images are shown for the three treatment groups in Figure 5A that display the cumulated number of sparks recorded in every 16 × 16 pixel regions of the fiber. Under control conditions only few active positions existed while during depolarization and in the presence of caffeine the events seemed to appear more equally spread along the fiber. The distribution of spark numbers among dedicated regions (16 × 16 pixel mesh) was compared to the Poisson distribution for all three treatments (Figure 5B). Since the theoretical Poisson functions calculated with the λ equal to the average of the event number was almost identical to the Poisson functions fitted to the measured values, our result suggests that both depolarization and caffeine treatment increased the frequency of the events evenly along the fiber without creating spatial clusters or ‘hot spots’ identified by concentrated activity specific to each conditions (Figure 5A).

**FIGURE 6:**
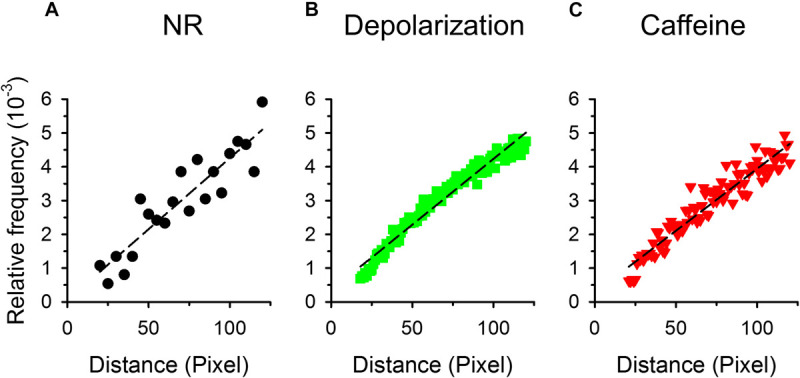
Distribution of the distances between the centers of two sparks. For each spark concentric squares were constructed around the center of the selected spark. The number of sparks with centers falling onto the side of the given square were then counted. The relative frequency of these neighboring sparks are presented for control conditions **(A)**, for depolarized **(B)**, and caffeine-treated fibers **(C)**. Note that in this representation the number of pixels increases linearly with the length of the side of the square, thus if sparks appear randomly a linearly increasing distribution is expected. To avoid the problem of bringing pixels from outside of the fiber into the counting 1./only those sparks were selected that lay close to the center of the fiber and 2./the side of the square was restricted to being less the 120 pixels (appr. 25 μm). Thus in the panels **(A–C)** the ordinate is in pixels. Dashed lines represent least-squares fits of a straight line to the data points in each graph with R^2^ being 0.829, 0.964, and 0.918, for panels **(A–C)**, respectively.

Based on the spatial cluster analysis we hypothesized that sparks were randomly distributed within the cell, therefore the distance distribution of the center-to-center for each spark pair would follow a linear function. Figure 6 presents the distances between all spark pairs on each frame for all images recorded. Only distances where one of the sparks was in the center of the respective fiber were considered in order to compensate for the finite size of the fiber. Furthermore, distances that were less than the radius of the given fiber were calculated. It should also be noted that events with centers closer than two FWHM could not be separated by the automated detection method (as their “contiguous areas” overlap and thus they are detected as a single, but large event; see Methods) thus only those sparks were considered where the centers were at least 15, 17, and 20 pixels (control, depolarization and caffeine, respectively) apart. Concentric squares (grid distance) were used instead of circles so as not to have any bias because of the grid pattern of pixels. Under control conditions (Figure 6A), on depolarized fibers (Figure 6B) and even in the presence of caffeine (Figure 6C) the data follow a close to linear function, as demonstrated by the superimposed dashed lines, confirming the random distribution of the events.

### Relative Position of Sparks to Z-Lines and Other Events

**FIGURE 7:**
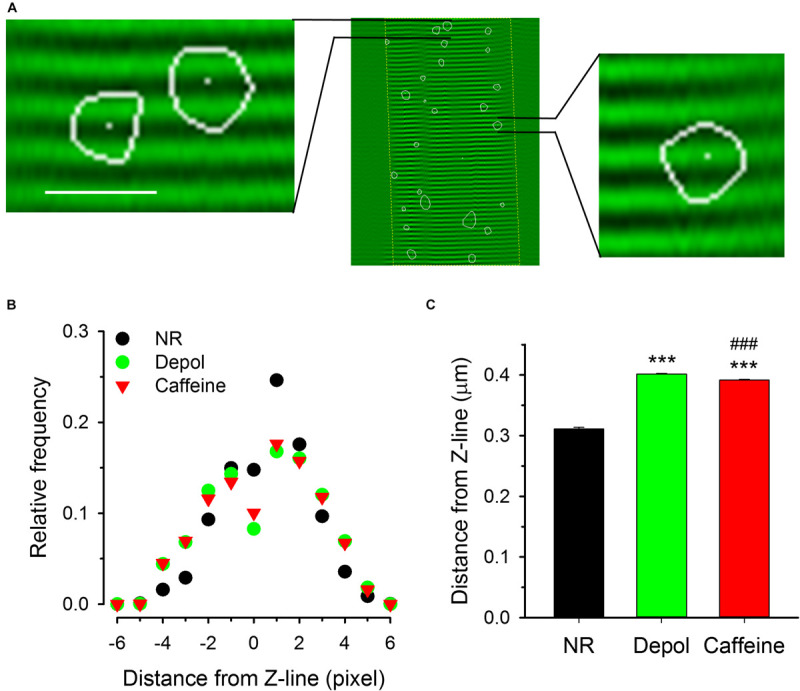
Localization of spark centers relative to the Z-line. **(A)** Two samples from an image showing the fiber structure superimposed with sparks (indicated by the areas encircled by the white lines). White dots present the position of the center of the sparks. **(B)** Distribution histograms of the distance between center of sparks and the nearest Z-line for control conditions (NR, black circles), on depolarized fibers (green squares), and in the presence of caffeine (red triangles). Note that the distributions are essentially symmetric to the Z-line. **(C)** Mean distances of spark centers under the three conditions. Both the depolarization and the presence of caffeine shifted the center of the spark to positions further away from the Z-line as compared to the control conditions. Scale in panel A corresponds to 40 μm for the middle image and 4.3 μm for the enlarged images on left and right side. The middle image was recorded with 30 FPS. Here and in subsequent figures *** denotes significant difference versus NR at *p* < 0.001, ^###^ denotes significant difference vs. Depol at *p* < 0.001.

The determination of fiber structure during the analysis of images allowed us to examine the positions of sparks relative to the Z-lines. Figure 7A shows the position and orientation of Z-lines with superimposed sparks (the outlines of sparks are marked with white curves in the enlarged images). The histogram in Figure 7B presents the distribution of the distances of the position of the pixel with the highest intensity for each spark from the nearest Z-line. Under control conditions the average distance was calculated to be 0.311 ± 0.003 μm, which was increased significantly either by depolarization (0.401 ± 0.001 μm) or caffeine (0.391 ± 0.002 μm) treatment (Figure 7C).

### Spatial Morphology of Sparks

**FIGURE 8:**
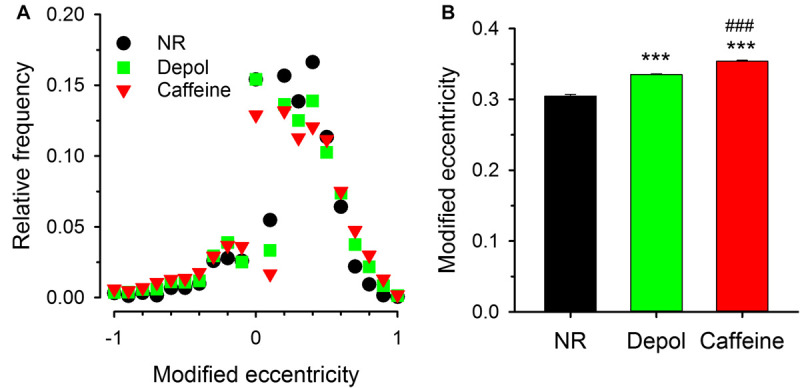
Modified eccentricity of the sparks **(A)**. Distribution of the modified eccentricity calculated using Eqn. 2 for control conditions (NR, black circles), for depolarized fibers (green squares), and in the presence of caffeine (red triangles). Note that albeit negative values were present, the distribution is not symmetric, it is shifted toward positive values indicating that FWHM-X was, in most cases, greater than FWHM-Y. **(B)** Average modified eccentricity for the three experimental conditions calculated using only the positive values from panel **(A)**. Note that both the depolarization and caffeine treatment increased the modified eccentricity, and, furthermore, its value was far greater even under control conditions than what is expected from the skewing introduced by the scanning.

To address the spatial orientation of sparks relative to the Z-lines FWHM-X and FWH-Y were measured under all experimental conditions and the modified eccentricity was calculated. As demonstrated in Figure 8A, the modified eccentricity showed an asymmetric distribution both under control conditions, on depolarized fibers, and in the presence of caffeine, indicating that FWHM-X was greater than FWHM-Y for most of the events detected. It is also clear from the histogram that the modified eccentricity was most frequent found between 0.30 and 0.42. This value is significantly greater than that expected to be introduced by scanning (see Supplementary Figure 6). Furthermore, larger modified eccentricity values appeared with greater frequency in depolarized as well as in caffeine treated cells. This is reflected in the mean values of modified eccentricity, as this mean was significantly greater for both the depolarized condition and the caffeine treatment (Figure 8B). Importantly, the asymmetric shape of the events suggests that either the source is extended or the diffusion is faster in the X direction, or both.

### Travelling Sparks

**FIGURE 9:**
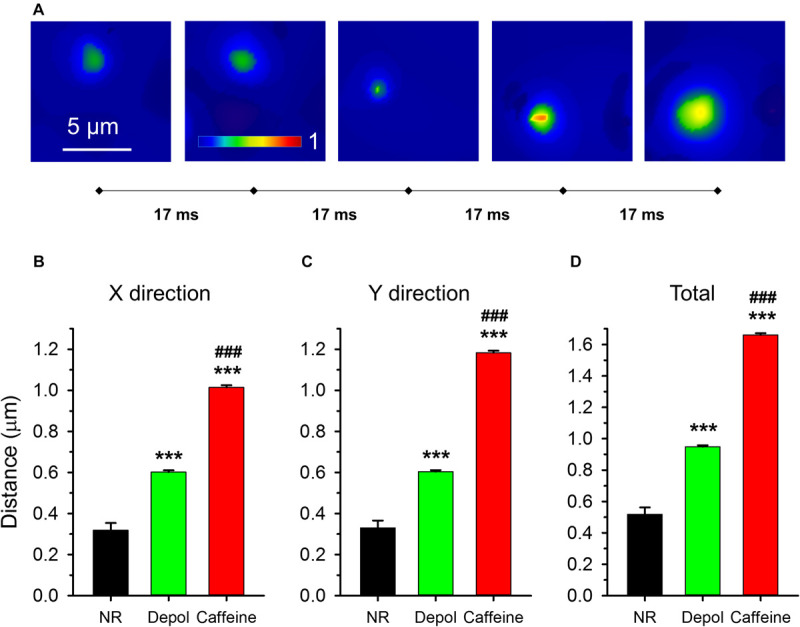
Traveling events on consecutive x-y images. **(A)** Five consecutive F/F_0_ images with the traveling sparks identified. The sampling rate was 17 ms/image (60 FPS). **(B–D)** Averages of distances traveled. **(B,C)** report distances in X and Y direction, respectively, while panel **(D)** presents the total distance traveled. The numbers of events analyzed are presented in Table 2.

Most sparks were visible only in one frame even when detected with high speed confocal microscopy. For sparks that were present in more than one consecutive frames (Figure 9A; Supplementary Video 2, 3), the distance traveled by the center of the spark between two frames was calculated (Figures 9B–D). Note that a traveling spark indicates the presence of calcium-induced opening of RyR(s) that didn’t get activated first at the beginning of the event.

While under control conditions only 1.5% of events appeared in two consecutive images, the probability was higher in depolarized fibers (9.8%) and even higher after caffeine treatment (29.7%). Sparks traveled significantly longer distances after caffeine treatment (1.66 ± 0.01 μm, *n* = 7416) than in depolarized fibers (0.95 ± 0.01 μm, *n* = 4247) which, nonetheless, was still significantly longer than under control conditions (0.52 ± 0.01 μm, *n* = 94; Figure 9D). While under control conditions and during depolarization the distance traveled by the events was the same in X and Y directions, in the caffeine-treated cells sparks traveled significantly longer distances especially in the Y direction (*p* < 0.001; Figures 9B,C). These results indicate that depolarization and caffeine treatment “primed” the release sites for subsequent activation by calcium.

## Discussion

In the past 15 years several automatic Ca^2+^ spark analyzer programs were developed. Some of them used Wavelet transformation methods (von Wegner et al., 2006; Szabo et al., 2010, Prada et al., 2018). Others employed noise thresholding (Picht et al., 2007), statistical analysis (Bányász et al., 2007), or pixel-by-pixel method (Illaste et al., 2019; Tian et al., 2019) to capture and analyze local calcium events.

In this study we showed that our Wavelet based automatic analysis method was suitable to reveal the spatial and temporal characteristics of calcium sparks detected via high-speed confocal microscopy. The program not only captured the calcium release events but was capable of identifying constant cell structures, like Z-lines, and calculate the distance between these structures and the detected sparks. Furthermore, it could calculate the distance between sparks and follow the movement of events appearing in consecutive images. These new features allowed us to describe the behavior of localized elementary calcium release more precisely, perform high throughput data analysis. It also gave us the opportunity to raise hypotheses on the role of the two types of RyRs present in frog skeletal muscle.

RyRα and RyRβ are present in nearly equal amounts in frog skeletal muscle, and are homologs of RyR1 and RyR3 of mammalian muscles (Ogawa et al., 2002). The depolarization-induced Ca^2+^ release is believed to be mediated by RyRα because of its similarity to RyR1 (Oyamada et al., 1994) and its location adjacent to DHPRs. While calcium-induced calcium release works with both types of RyRs in frog skeletal muscle, its activity on RyRα may have 20-fold lower than that on RyRβ (Murayama and Ogawa, 2001). From this, one can hypothesize that the two isoforms of RyR may have distinct roles in calcium release. We successfully showed that sparks detected on depolarized or caffeine treated frog muscle fibers have distinct spatiotemporal parameters and amplitude distribution in our experiments.

We found that sparks appeared at a higher frequency in both depolarized and caffeine treated fibers as compared to control. Importantly, although both the depolarization and caffeine increased both the signal mass of the sparks – i.e., the amount of calcium released during the event – and their frequency, this was not always the case when applied together. At greater depolarizations albeit the frequency was further increased, the signal mass, due to a drop in both the amplitude and the spatial spread, decreased dramatically. This can be reconciled if one assumes that the large number of events leads to a partial depletion of calcium in the terminal cisternae of the SR. Indeed, if the rate of calcium release exceeds the uptake rate of the SR calcium pump one expects a depletion of calcium in the SR. However, in this case the cytosolic calcium concentration should increase as the released but not reuptake calcium should appear in the intracellular space. Although Fluo-4 is not suitable to allow a precise calculation of the exact calcium concentration, the overall increase in fluorescence seen at greater depolarizations supports this idea.

The analysis of FWHM on depolarized fibers treated with caffeine (Supplementary Figure 7) suggests that the two interventions exerted their effect independent of one another. We also observed widening of the spatial spread and shifting of the center of the spark further away from the Z-line (Figure 7C and Supplementary Figure 9A). In this framework under control conditions a small group of RyRα-s open under a strict control of DHPRs and depolarization recruits additional RyRα-s. The calcium thus released can then albeit to a small extent activate additional release channels via CICR. The addition of caffeine primes the release channels to the activating calcium making CICR more probable. As RyRβ-s are more sensitive to caffeine than RyRα (see e.g., Murayama and Ogawa, 2001; Kashiyama et al., 2010) calcium sparks in the presence of caffeine are likely to be generated more by RyRβ than by RyRα. This is reflected in our finding that sparks in the presence of caffeine appear with greater amplitude and frequency (Table 2). RyR-deficient myotubes expressing RyRα have uniform and sustained calcium release, whereas cells expressing RyRβ have regenerative Ca^2+^ release events (waves and oscillations; Kashiyama et al., 2010). Examining the distribution of amplitudes and FWHMs of the sparks revealed that under control conditions the spontaneously generated calcium sparks were initiated predominantly by RyRα since the distribution of their parameters was similar to those on depolarized fibers (Figures 4B–D). An important finding of our study was that the signal mass of sparks (representing the amount of calcium released) was only slightly increased by depolarization, but multiplied after caffeine treatment (Table 2).

The high number of events allowed us to statistically analyze the appearance of sparks macroscopically in a fiber. One important finding was that there were no “hot spots” where more sparks would develop than at other places in intact frog skeletal muscle fibers. The appearance of sparks in the whole fiber followed the Poison distribution in all cases examined (Figure 5). Similar results were found by Klein et al. (1996) during depolarization in also frog muscle fibers. This suggests that the events arose following a random process independently from depolarization and the presence of caffeine. The microscopic analysis of event position lead to the same conclusion. Interspark distances are close to linearly distributed on depolarized or caffeine treated muscle fibers indicating that sparks form independently from one another (Figure 6). Nevertheless, a slight deviation from this linearity can be observed at short distances on depolarized fibers and in the presence of caffeine. It was hypothesed by Stern et al. (2013) that calcium release from one couplon could influence its neighbors in several ways: (1) SR calcium can travel to other couplons by diffusion within the SR lumen; (2) cytosolic calcium can diffuse to activate or influence RyRs; and (3) SR calcium can be increased by SERCA uptake following the diffusion in the cytosol.

Calcium sparks were asymmetric and wider in the direction parallel to the axis of the muscle fibers. The events exhibited some eccentricity (Figure 8, Supplementary Figures 6, 9B) comparable to that what was reported for the RyR cluster shape in cardiac myocytes (Galice et al., 2018). This behavior of sparks was independent of the depolarization and caffeine treatment suggesting the idea that the size of RyR cluster creating a sparks was greater than in control but similar in the two investigated conditions. We have to underline that the difference between the FWHM values in the X and in the Y direction did not result from the non-instantaneous scanning of the frame (Supplementary Figure 6A). Taking the value for the speed of Ca^2+^ diffusion in the cytosol (0.73 μm/ms; Donahue and Abercrombie, 1987) and the speed of scanning (3.19 μm/ms, calculated as 106 μm/512/0.065 ms from 65 μs/line, 512 lines/frame, 106 μm/frame scanning parameters) one can conclude that the speed of diffusion is around one quarter of the scanning. This would result in a deformed spot of release (see Supplementary Figure 6A) and contribute to the eccentricity of events. The theoretical eccentricity calculated from the two speeds (diffusion divided by scanning) was 0.23 in our case. Eccentricity, however, did not take the orientation of the spark relative to that of the fiber into account. We have, thus introduced a modified eccentricity to keep this spatial information. From the calculations the eccentricity introduced by the scanning and represented by the value of the modified eccentricity was much smaller than those observed under either condition (see Supplementary Figure 5). This indicates that either the source was extended or the diffusion was not homogenous in all directions, or both. Furthermore, as both the depolarization and caffeine increased the value of modified eccentricity and neither is likely to alter the diffusion of calcium in the cytosol, one must conclude that they affected the size of the source. This is substantiated by the finding that both interventions increased the signal mass, i.e., the amount of calcium released, in an event.

The detailed examination of consecutive images provided that only a minority (1.5%) of sparks could “travel” from their origin by around half μm under control conditions. Depolarization doubled and caffeine tripled this distance in average (Figure 9D) and the number of traveling sparks increased dramatically in both cases (∼10% and ∼30% of all events, respectively). Similar observation was obtained after the application of the special calcium chelator TPEN (Sztretye et al., 2009). All these interventions, depolarization and caffeine in this report and TPEN in that of Sztretye et al. (2009) have one feature in common. Namely, they prime RyRs for oncoming activating stimuli, in our case the calcium that diffuses from a neighboring release site. The observation that caffeine promoted longer distances of traveling than the depolarization did argue in favor of the hypothesis that the latter involved RyRs located further away from the original release site.

## Conclusion

In this study we have analyzed a vast number of calcium sparks from intact frog skeletal muscles under control condition, during subthreshold depolarizations and in the presence of caffeine. We have introduced a method that enabled us to obtain spatial information of spark morphology and location relative to the orientation of the fiber axis and the Z-line. Understanding the role of CICR in the generation of spontaneous calcium sparks could help in revealing the mechanisms underlying the appearance of such events under pathological conditions and during regeneration in mammalian skeletal muscle. Furthermore, the role RyRα and RyRβ play in the generation of spontaneous calcium sparks should shed light on the interaction of RyR1 and RyR3 in those mammalian muscle fibers where RyR3 is expressed in greater amounts.

## Data Availability Statement

The raw data supporting the conclusions of this article will be made available by the authors, without undue reservation.

## Ethics Statement

The animal study was reviewed and approved by University of Maryland Institutional Animal Care and Use Committee.

## Author Contributions

HC and MS designed the experiments. HC participated in the confocal microscopy spark experiments. JV wrote the image analyzer program. BD, HC, LC, and PS wrote the manuscript. DB, JV, and PS did the statistical analysis and prepared the figures. All authors contributed to discussion, and reviewed/edited the manuscript.

## Conflict of Interest

The authors declare that the research was conducted in the absence of any commercial or financial relationships that could be construed as a potential conflict of interest.
