# Supplementary figures and images for: High Time Resolution Analysis of Voltage-Dependent and Voltage-Independent Calcium Sparks in Frog Skeletal Muscle Fibers
Source: Front Physiol. 2020 Dec 15;11:599822. doi: 10.3389/fphys.2020.599822 (PMC7769825; doi:10.3389/fphys.2020.599822)

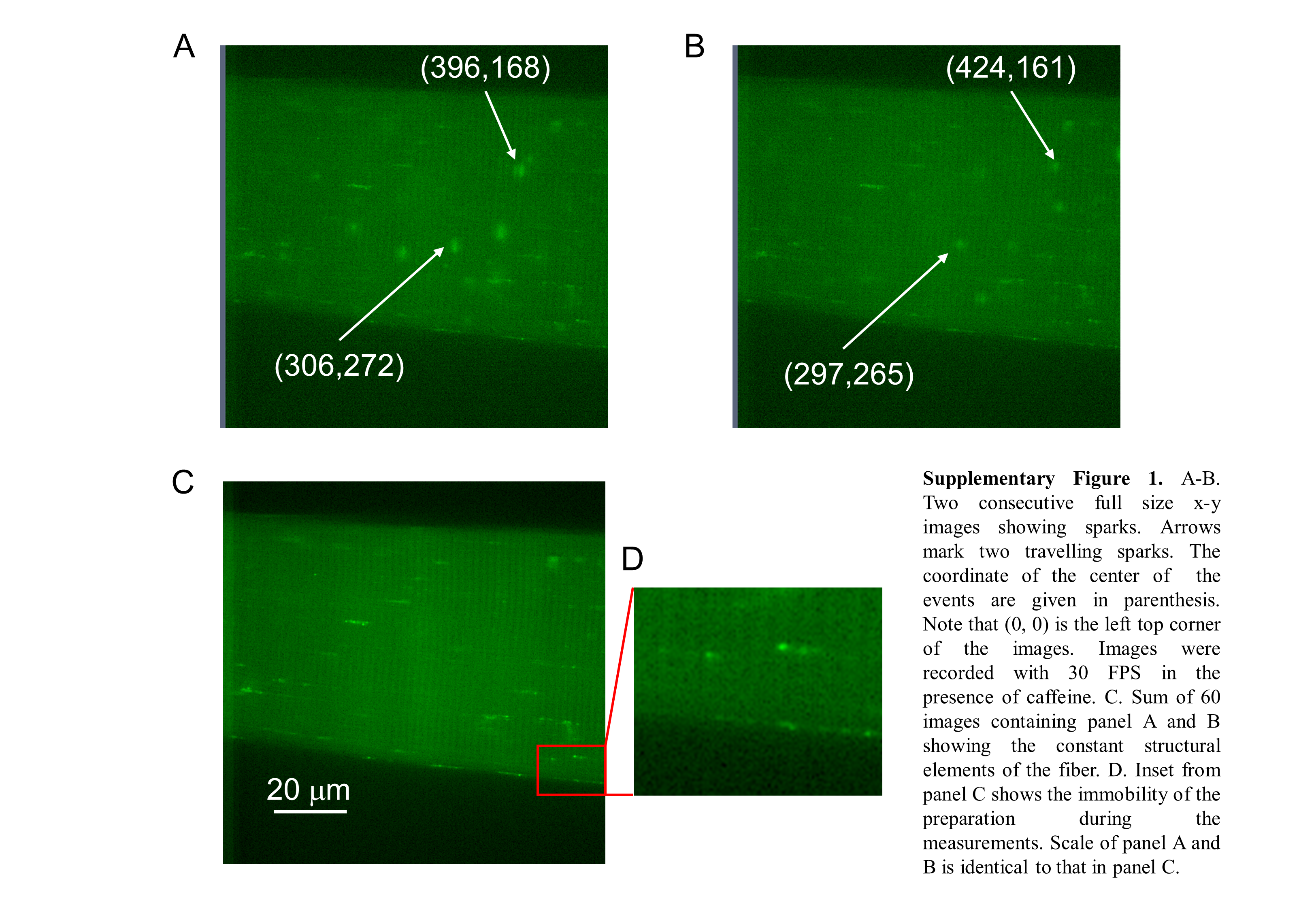

Supplement: Supplementary file 4 [file Image_1.TIF]

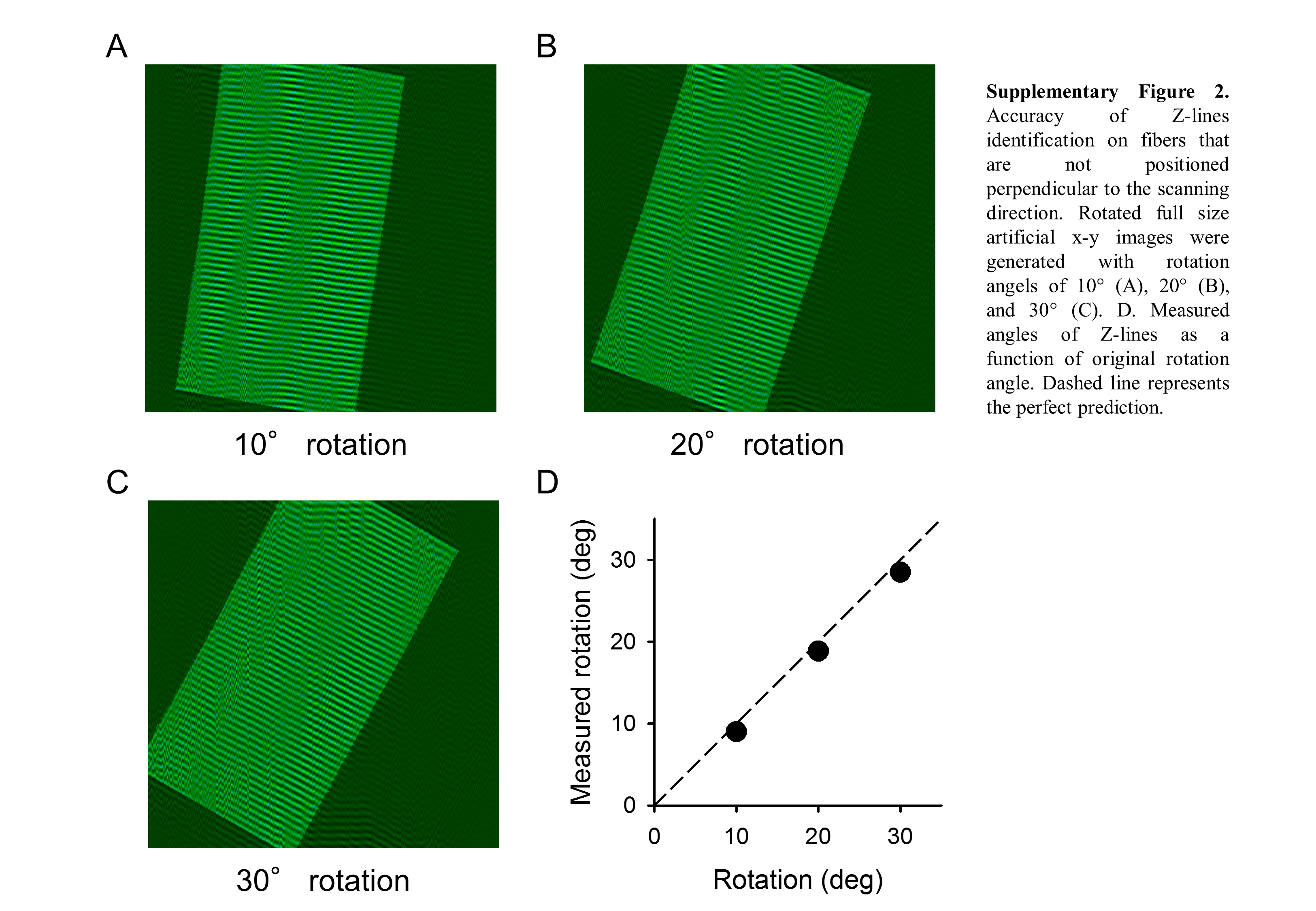

Supplement: Supplementary file 5 [file Image_2.TIF]

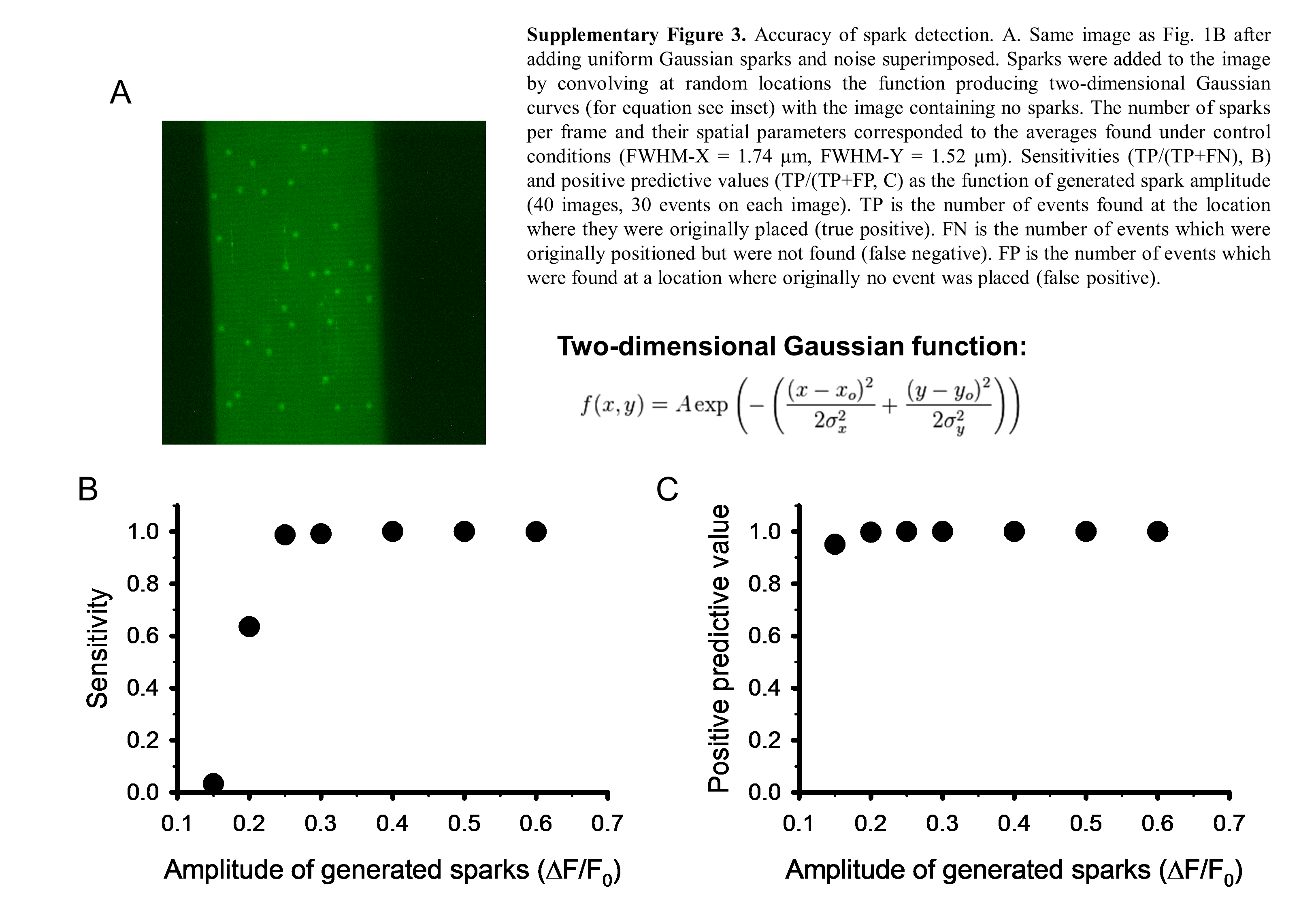

Supplement: Supplementary file 6 [file Image_3.TIF]

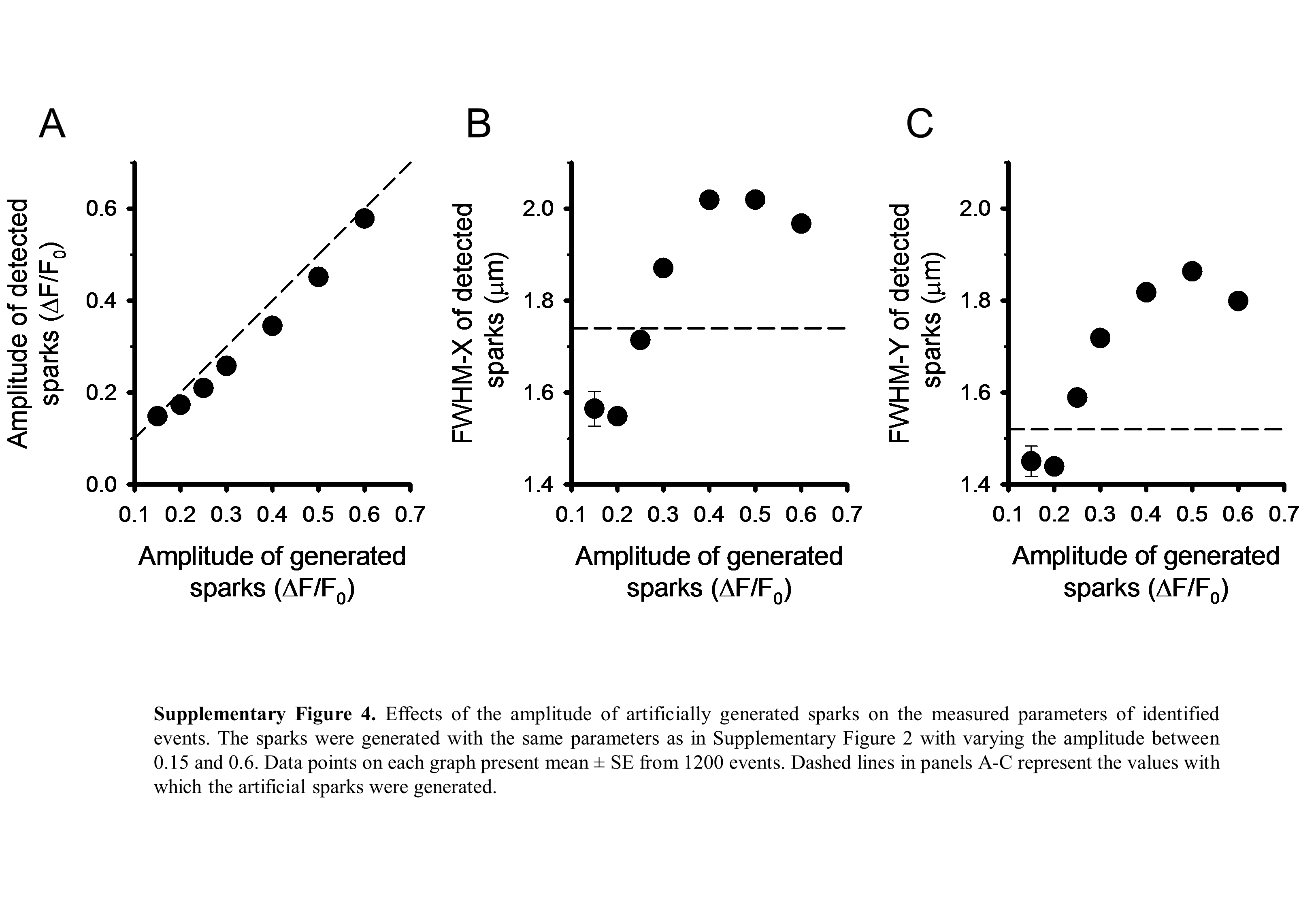

Supplement: Supplementary file 7 [file Image_4.TIF]

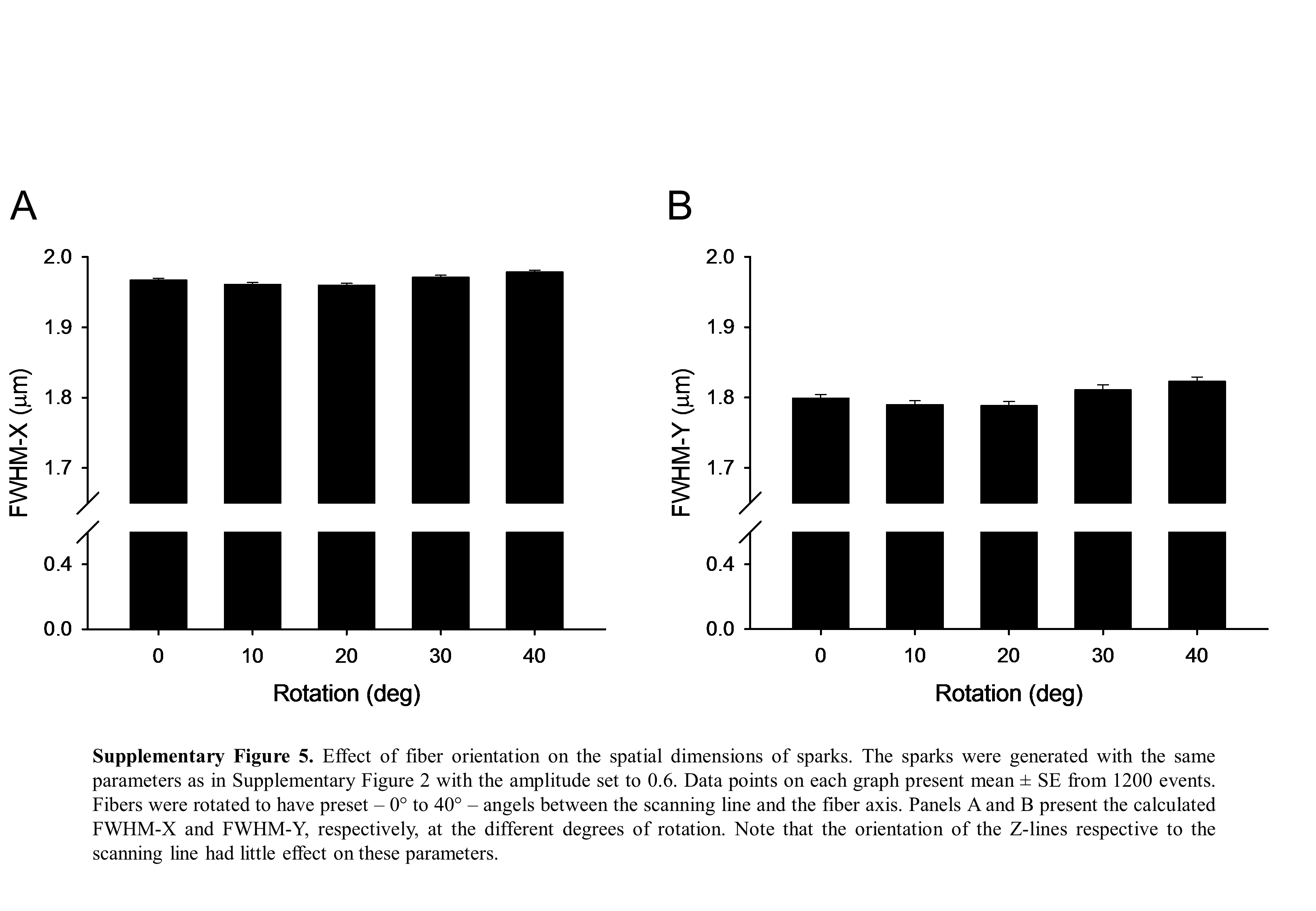

Supplement: Supplementary file 8 [file Image_5.TIF]

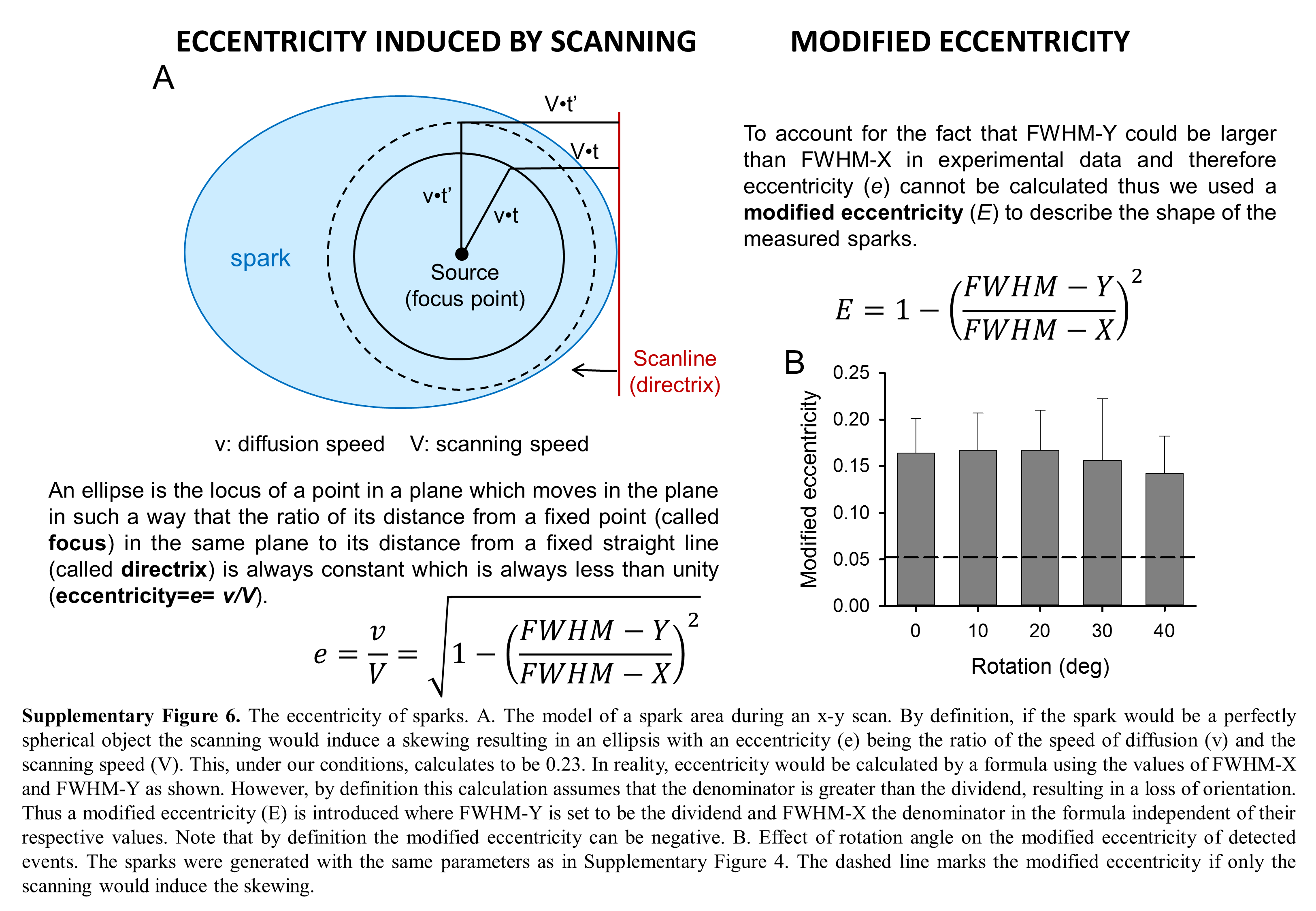

Supplement: Supplementary file 9 [file Image_6.TIF]

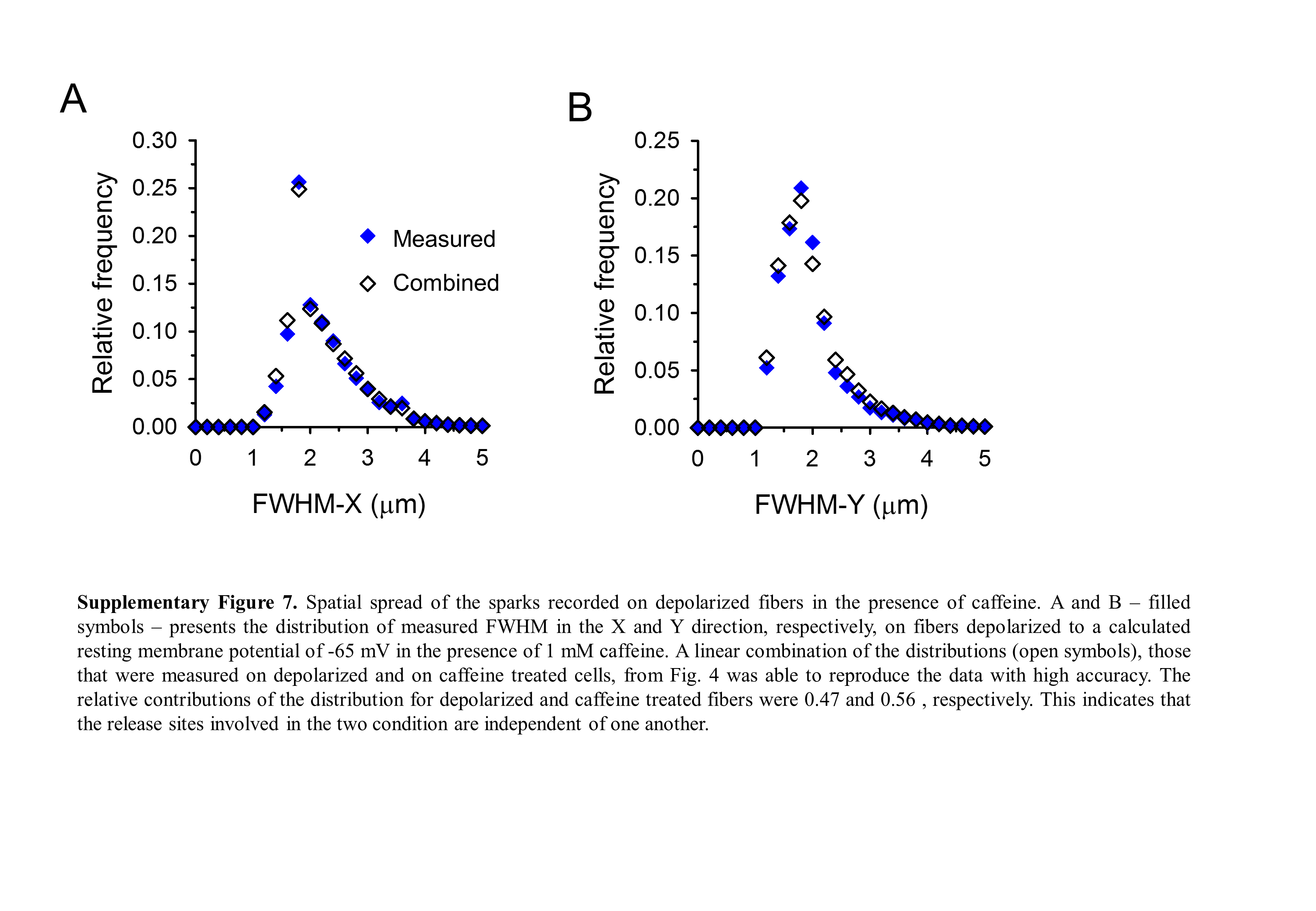

Supplement: Supplementary file 10 [file Image_7.TIF]

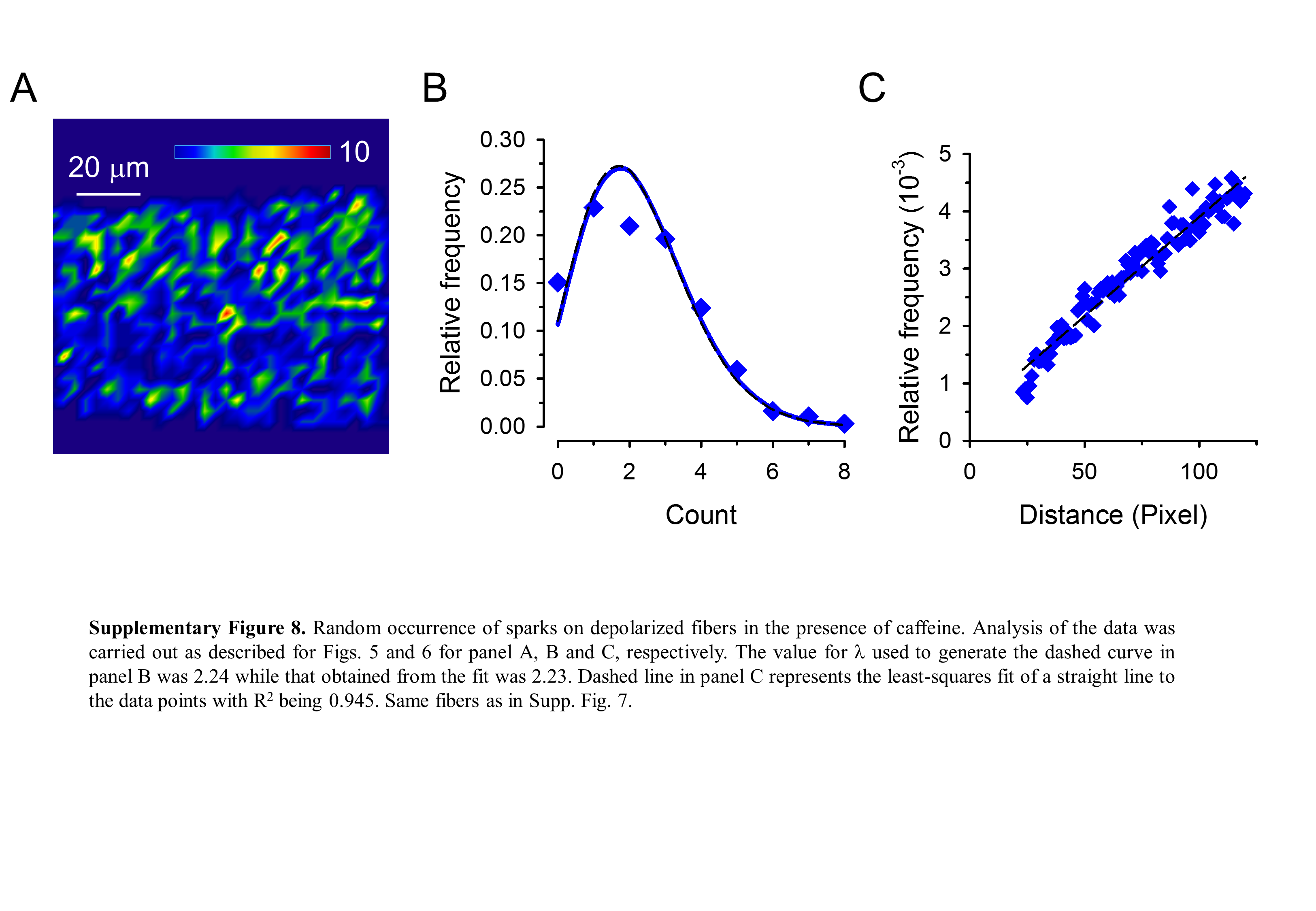

Supplement: Supplementary file 11 [file Image_8.tif]

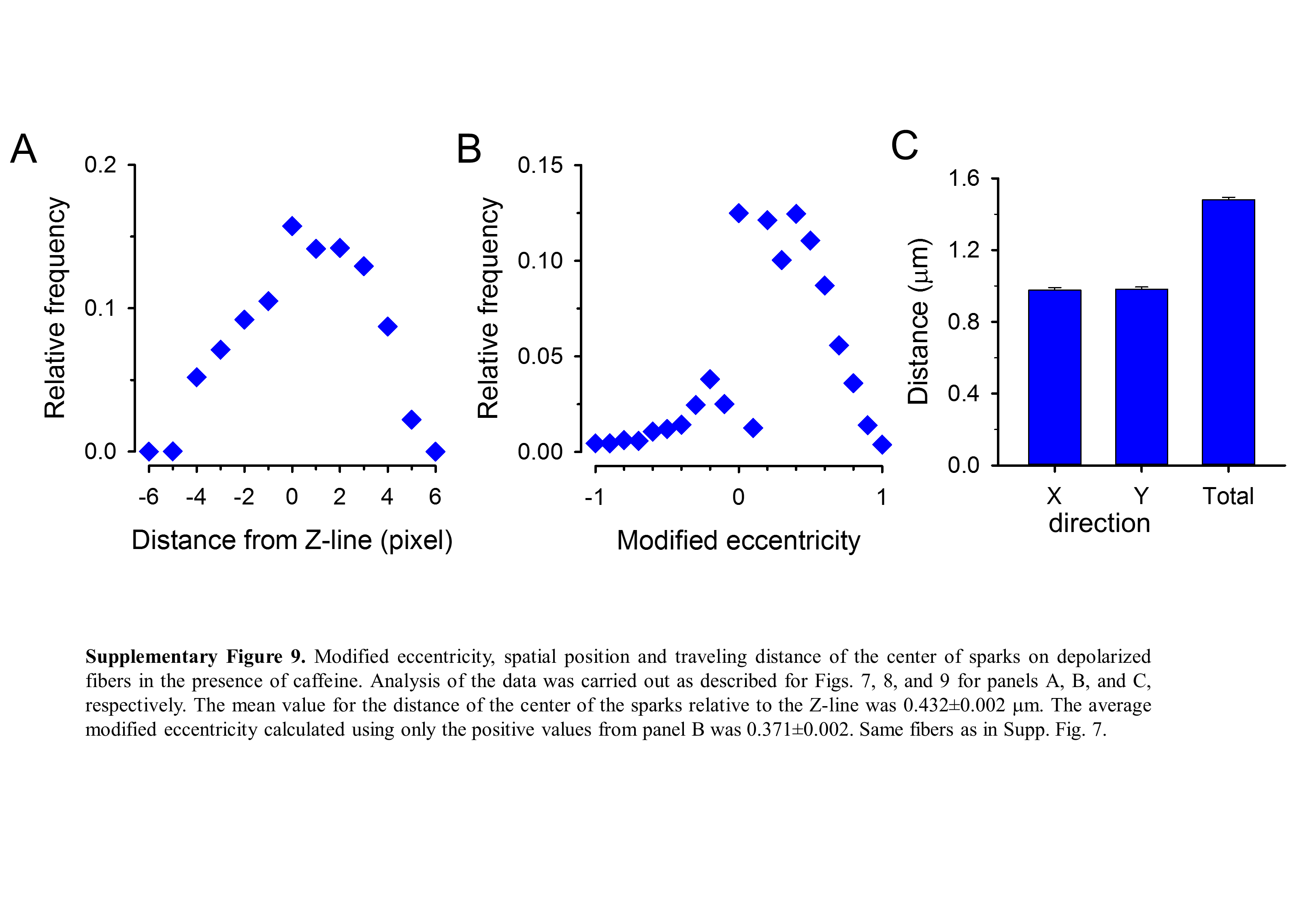

Supplement: Supplementary file 12 [file Image_9.tif]
